# The role of epicardial adipose tissue dysfunction in cardiovascular diseases: an overview of pathophysiology, evaluation, and management

**DOI:** 10.3389/fendo.2023.1167952

**Published:** 2023-05-16

**Authors:** Cheng Li, Xinyu Liu, Binay Kumar Adhikari, Liping Chen, Wenyun Liu, Yonggang Wang, Huimao Zhang

**Affiliations:** ^1^ Department of Cardiovascular Center, The First Hospital of Jilin University, Changchun, Jilin, China; ^2^ School of Basic Medical Sciences, Changchun University of Chinese Medicine, Changchun, Jilin, China; ^3^ Department of Cardiology, Nepal Armed Police Force Hospital, Kathmandu, Nepal; ^4^ Department of Echocardiography, Cardiovascular Center, The First Hospital of Jilin University, Changchun, Jilin, China; ^5^ Department of Radiology, The First Hospital of Jilin University, Jilin Provincial Key Laboratory of Medical Imaging and Big Data, Changchun, Jilin, China

**Keywords:** epicardial adipose tissue, obesity, cardiovascular diseases, cardiac imaging, management

## Abstract

In recent decades, the epicardial adipose tissue (EAT) has been at the forefront of scientific research because of its diverse role in the pathogenesis of cardiovascular diseases (CVDs). EAT lies between the myocardium and the visceral pericardium. The same microcirculation exists both in the epicardial fat and the myocardium. Under physiological circumstances, EAT serves as cushion and protects coronary arteries and myocardium from violent distortion and impact. In addition, EAT acts as an energy lipid source, thermoregulator, and endocrine organ. Under pathological conditions, EAT dysfunction promotes various CVDs progression in several ways. It seems that various secretions of the epicardial fat are responsible for myocardial metabolic disturbances and, finally, leads to CVDs. Therefore, EAT might be an early predictor of CVDs. Furthermore, different non-invasive imaging techniques have been proposed to identify and assess EAT as an important parameter to stratify the CVD risk. We also present the potential therapeutic possibilities aiming at modifying the function of EAT. This paper aims to provide overview of the potential role of EAT in CVDs, discuss different imaging techniques to assess EAT, and provide potential therapeutic options for EAT. Hence, EAT may represent as a potential predictor and a novel therapeutic target for management of CVDs in the future.

## 1 Introduction

Cardiovascular disease (CVD) is a considerable health condition that affects millions of individuals all over the world. To date, many risk factors are associated with the increasing incidence of CVDs. Among them, obesity has gained wide scientific interest. Obesity is closely associated with many other cardiovascular disease risk factors such as hypertension, dyslipidemia, metabolic syndrome, and diabetes mellitus. It is well-established that increased adiposity releases plenty of inflammatory cytokines that lead to a low-grade inflammatory microenvironment, endothelial dysfunction, and oxidative stress, and finally results in several CVDs (1, 2). The epicardial adipose tissue (EAT) is the visceral fat that deposits between the visceral pericardium and the myocardium and has direct contact with the myocardium and coronary artery (3). It usually presents as a white adipose tissue, but it also displays brown or beige fat-like features (4). Physiologically, EAT serves as thermoregulator and provides energy to the myocardium. Furthermore, EAT displays as an endocrine organ with metabolic activities and secretes bioactive molecules that affect the heart and coronary arteries *via* paracrine or vasocrine effects (5, 6). In recent years, evidence has shown that EAT is associated with CVDs. Therefore, different non-invasive imaging techniques have been proposed to identify and assess EAT to evaluate the risk of CVDs.

There are mainly three non-invasive imaging techniques that are used to evaluate EAT. First, echocardiography is used to evaluate EAT, which measures two-dimensional EAT thickness. It is an inexpensive, readily available, fairly accurate, and reproducible technique. Cardiac computed tomography (CCT) and cardiac magnetic resonance (CMR) imaging allow for three-dimensional EAT estimation. The former has higher space resolution and reproducibility for fat quantification, but it has limitations of radiation exposure and complex manual segmentation. However, the latter has no radiation exposure, but it is limited by space resolution, reproducibility, and higher cost. CMR is also difficult to perform in obese patients.

In this review, we summarize anatomical, physiological, and pathophysiological characteristics of EAT and focus on the potential role of EAT in CVDs and discuss different imaging techniques to assess EAT. In recent years, several papers have shown that EAT measurement *via* non-invasive imaging techniques serves as an important diagnostic tool to assess cardiovascular risks. Therefore, EAT may be a potential biomarker to monitor CVDs and their complications.

## 2 Epicardial adipose tissue: anatomy and physiology

### 2.1 Anatomy

**Figure 1:**
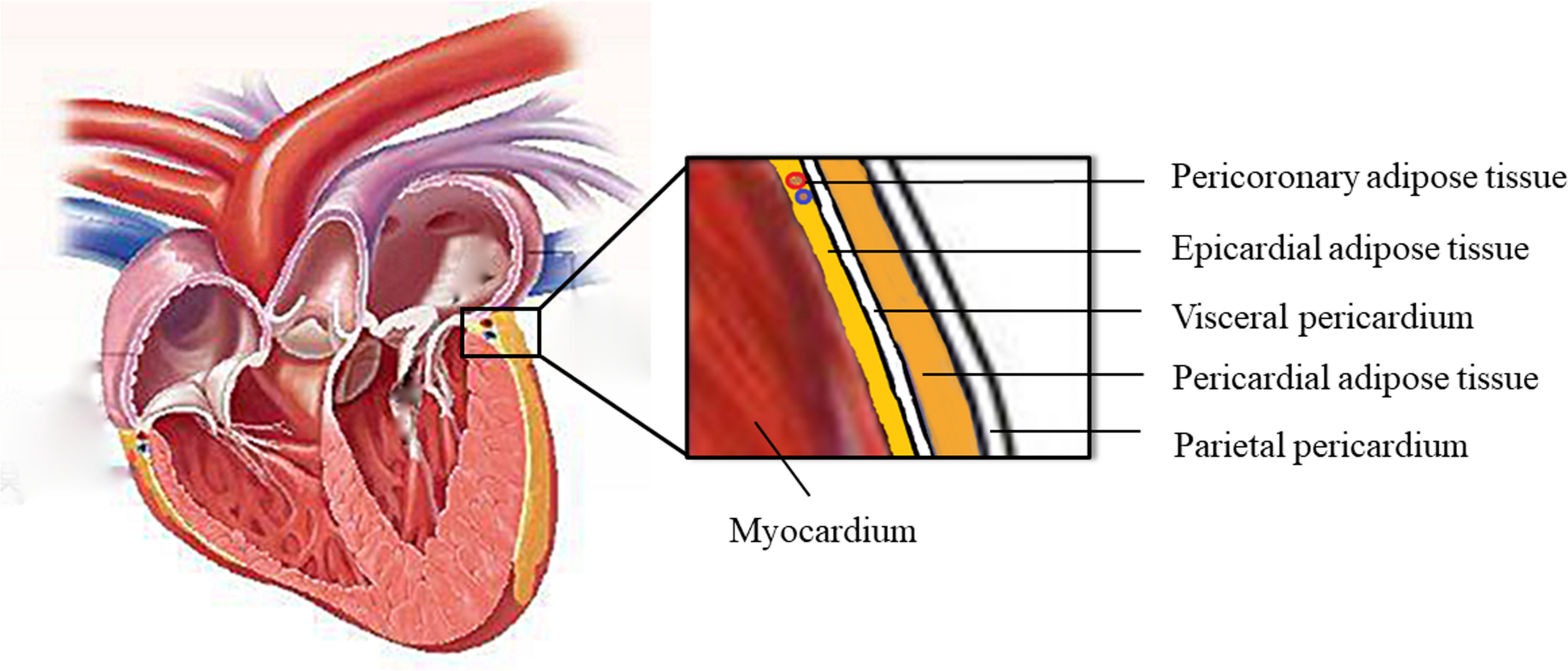
The anatomy of epicardial adipose tissue.

The adipose tissue surrounding the heart can be divided into EAT, pericardial adipose tissue, paracardial adipose tissue, and perivascular adipose tissue (**Figure 1**). EAT lies between the myocardium and visceral pericardium and is made up of adipocytes, ganglia, nerves, and inflammatory, stromovascular, and immune cell (6). The pericardial adipose tissue (PAT) consists of epicardial and paracardial fat depots (7). The EAT and PAT are the entire pericardial fat. They have different embryological origins but share similar morphological features. EAT is derived from the splanchnopleuric mesoderm, and PAT is derived from the thoracic mesoderm (7).

EAT makes up 20% of the cardiac mass and covers 80% of the cardiac surface under normal physiological condition. It is non-homogeneously distributed around the heart (6, 8). EAT is mostly localized at the cardiac base and apex, in the atrioventricular and interventricular grooves, and around the coronary arteries. It is thicker around the right ventricle than around the left ventricle. In general, EAT can be differentiated into peri-coronary and peri-myocardial EAT. The former is located directly around or on the coronary artery adventitia; the latter is located just over the myocardium and is in direct contact with the myocardium (9). Vascular supply by coronary arteries in EAT forms part of the perivascular adventitia (10). It is thought to play a protective mechanical role against the tension and twist of an arterial pulse (11, 12). The increased EAT might result in cardiac disorders with increased arrhythmogenicity (13). It is hypothesized that EAT increases fatty infiltrates in the proximity of myocytes that leads to structural remodeling and abnormal impulse generation, which contributes to cardiac arrhythmias (13).

### 2.2 Physiology

#### 2.2.1 Physical protective barrier and energy fat source

The epicardial fat surrounds the coronary arteries and myocardium; hence, EAT is considered to act as a buffering system in normal physiological conditions. It protects the heart and coronary arteries from mechanical deformation and facilitates vascular remodeling (14). In addition, EAT has thermogenic function that protects the heart from hypothermia. The increased thermogenic potential is due to brown adipocytes in EAT. Since EAT is more lipolytic than other adipose tissue depots, it releases abundant free fatty acids (FFAs) during high energy demand period, which is the main source of energy for the myocardium (14–16). In addition, EAT is present close to the myocardium and acts as a buffer to protect the heart from exposure to excessively high FFAs and lipotoxicity (17, 18).

#### 2.2.2 Adipose tissue properties

Based on embryological, histological, and functional aspects, adipose tissue can be divided into two major groups: white and brown adipose tissue. The former has relatively few mitochondria and a single big lipid droplet, while the latter has multiple small lipid droplets and abundant mitochondria (19). EAT is basically a white adipose tissue but also has brown and beige fat-like features. It releases many mediators through expressing thermogenic genes related to brown and beige adipose tissues, such as tumor necrosis factor alpha (TNF-α), interleukin (IL)-1β, IL-1 receptor antagonist, IL-6, IL-8, IL-10, C-reactive protein (CRP), and plasminogen active inhibitor 1 (20). These factors may be involved in the communication between EAT and myocardial tissue through endocrine effect because they share the same capillary circulation (19). It is reported that EAT is twice as metabolically active as normal white adipose tissue, which is related to lipolysis and free fatty acid release. In addition, EAT has an increased capacity to release free fatty acids into the blood circulation and decreased glucose consumption compared to other adipose tissues (18). It also alters the bioavailability of adipokines and leads to adipocyte hypertrophy, tissue hypoxia, inflammation, and oxidative stress (7, 21). Brown fat generates heat in response to cold temperature and autonomic nervous system activation. Like brown fat, EAT also protects the myocardium from hypothermia.

#### 2.2.3 Endocrine organ

Besides acting as energy depot, EAT also serves as an endocrine organ that regulates the heart homeostasis. There are two classical interaction mechanisms between the myocardium and the EAT: vasocrine and paracrine. On the one hand, adipokines and FFAs, as vasocrine signaling molecules, are released from EAT that enters the vasa vasorum directly and are transported downstream into the arterial wall. On the other hand, EAT-derived adipokines diffuse in interstitial fluid that cross the vascular wall (adventitia, media, and intima), and finally interact with vasa vasorum, endothelia, and vascular smooth muscle cells of the coronary arteries (18, 22). However, extracellular vesicles, containing cytokines and microRNAs have been confirmed as new communication modes (23). FFAs are the main energy source of the heart. EAT secretes vasoactive products that regulate coronary arterial tone to facilitate the FFA influx. In addition, fatty acid binding protein-4, expressed by EAT, may participate in the intracellular transport of FFAs from EAT into the myocardium (24).

## 3 Cardiac imaging of EAT

### 3.1 Echocardiography

The advantages of echocardiography to measure EAT thickness include low cost and more convenient, accessible, and reproducible (**Table 1**). However, there are few limitations. It is operator dependent. EAT is located in some areas of the heart that cannot be visualized with the ultrasound. In addition, obese patients have poor acoustic window. Although EAT thickness is considered as a useful diagnostic tool, the normal value of EAT is still undetermined. Iacobellis et al. (25, 26) reported a transthoracic echocardiographic method of evaluating EAT thickness on the free wall of the right ventricle from both parasternal long- and short-axis views. They choose the right ventricle to measure EAT because it is considered as the thickest absolute epicardial fat layer (27), and parasternal long- and short-axis views allow the most accurate measurement of EAT on the right ventricle with optimal cursor beam orientation. In addition, they reported an average epicardial fat thickness of 7 mm in men and 6.5 mm in women for standard clinical references (28). Another study that enrolled 459 patients with Grade I and II essential hypertension demonstrated that patients with EAT thickness >7 mm exhibited higher left ventricular mass index, diastolic dysfunction, and increased carotid stiffness and intima-media thickness (29). In addition, Islas et al. reported that acute myocardial infarction patients with EAT >4 mm have worse left ventricular systolic function and have large infarct size. EAT >4 mm is an independent predictor of major adverse cardiovascular events at 5-year follow-up (30).

**Table 1:** Comparison among the main imaging techniques for the evaluation of EAT.

Imaging techniques	Echocardiography	Computed tomography imaging	Magnetic resonance imaging
Availability	readily available	not readily available	not readily available
Invasive	non-invasive	minimally invasive	minimally invasive
Cost	low	medium	high
Radiation	no	yes	no
Operator-dependent	yes	no	no
Definition	low	high	medium
Scan time	quick	quick	long
Patient limitation	severely obese	allergic to contrast media	claustrophobia
Attenuation quantification	no	yes	no
EAT thickness assessment	yes	yes	yes
EAT volume assessment	No	yes	yes
Coronary artery calcification	No	yes	no

In addition, Parisi et al. presented a novel method to measure EAT thickness at the level of the fold of Rindfleisch, a pericardial recess where the parietal pericardium does not exert a mechanical compression on visceral fat (31). Moreover, echo-EAT thickness showed a significant correlation with the CMR-EAT thickness, both measured at the Rindfleisch fold. Although echocardiography is convenient and reproducible, it cannot reflect the variability in EAT thickness or EAT volume accurately. Multidetector CT and cardiac MRI can provide a more accurate and volumetric quantification of EAT.

### 3.2 Cardiac computed tomography

CCT is another imaging modality used to measure EAT. Although CCT has high spatial resolution and provides three-dimensional view of the heart, it is costly and requires radiation exposure (**Table 1**). Currently, coronary CT angiography (CTA) provides an optimal method that enables the characterization of morphological changes in the pericoronary adipose tissue (PCAT) and simultaneous assessment of coronary atherosclerosis (32). CT attenuation of the adipose tissue reflects morphological derangements of adipocytes that are exposed to the effects of local vascular inflammation. EAT volume and density are considered as independent markers of adverse cardiometabolic risk that can be measured by CCT (33, 34). Additionally, increased EAT volume is a predictor of CVDs that include obstructive coronary stenosis, myocardial ischemia, and coronary syndromes (33, 35–37). However, the upper cutoff CT value of normal EAT remains undermined. Dey et al. reviewed literatures that reported different inclusion criteria with CT value varying between 125.0 and 139.4 cm^3^ for men and 119.0–125.0 cm^3^ for women (38). In addition, EAT density or attenuation has also been associated with CVDs. It is reported that EAT attenuation is associated with coronary artery calcification, acute myocardial infarction, and coronary adverse events (34, 39–41). PCAT is considered as a metric of local vascular inflammation. The widely accepted definition of PCAT by coronary CTA is voxels ranging from −190 to −30 Hounsfield units, with volume of interest that extends to an orthogonal distance equivalent to the diameter of the target vessel (42). Antonopoulos et al. presented a method to detect coronary inflammation by characterizing the changes in PCAT CT attenuation (43). They have demonstrated that the average attenuation of EAT is inversely related to adipogenic gene expression and adipocyte size in a large cohort of patients who have undergone cardiac surgery.

Nowadays, although manual segmentation of EAT quantification is the method of choice, it is operator dependent, time consuming, and not suitable for routine clinical practice. Thus, artificial intelligence that includes machine and deep learning received more attention to obtain fast, automatic, and reliable measures of EAT by CCT.

### 3.3 Cardiac magnetic resonance

CMR is now considered as the gold standard for measuring visceral adipose tissue (44–46). CMR provides excellent visualization of visceral and parietal pericardia. Cardiac imaging is not affected in patients with excess subcutaneous fat. It enables easy assessment and volumetric quantification of EAT. Although there is no use of radiation and contrast agents, CMR is expensive and time consuming. It is also difficult to perform in patients with claustrophobia (**Table 1**). Additionally, CMR can differentiate cardiac fat into EAT and paracardial fat.

MRI provides explicit parameters about EAT volume and mass by using the spin-echo sequence technique (2, 47). Manual contouring of EAT area at end-diastole during cardiac cycles provides precise quantification of EAT volume (47). A 3D-Dixon sequence (an electrocardiography triggered and respiratory navigator gated 3D-gradient echo pulse sequence was used for cardiac Dixon imaging) has been shown to be a reliable method for EAT quantification in studies (48). Dixon method separates fat and water signal *via* voxel intensity differences present between in- and opposed-phase MR images (48). Rami Homsi et al. (49) enrolled 34 healthy volunteers (22 men; BMI range, 14–42 kg/m(2); age range, 21–79 years) and measured parameters of pericardial and epicardial adipose volume (PAV, EAV) using a 3D-Dixon-based CMR approach. They found that the average EAV was 77.0± 55.3 ml, and PAV was 158.0 ± 126.4 ml; both were highly correlated. Therefore, they proposed a 3D-Dixon-based method that allows accurate measurement of cardiac fat volume and provides a valuable tool for cardiovascular risk stratification.

Moreover, CMR measures EAV, left ventricular compliance, pulse wave velocity, and other indicators simultaneously, which can evaluate aortic stiffness, myocardial strain, and fibrosis (50, 51). A combined measurement by CMR may support the evaluation of risk and prognosis of CVDs.

## 4 Epicardial adipose tissue: a new biomarker for cardiovascular disease risk assessment

**Figure 2:**
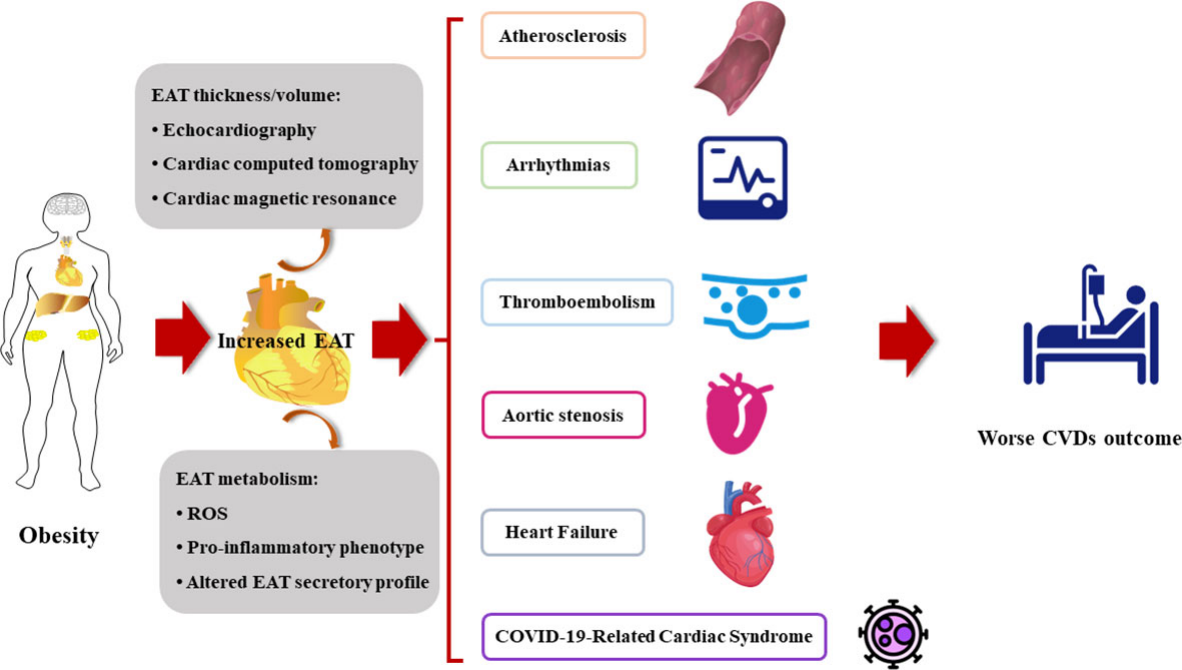
The increased EAT contributes to the onset and the poor prognosis of cardiovascular diseases. CVDs, cardiovascular diseases; EAT, epicardial adipose tissue; ROS, reactive oxygen species.

Evidence strongly supports the role of structural and functional changes of EAT in the pathogenesis of various cardiovascular diseases (**Figure 2**).

### 4.1 Atherosclerosis

Atherosclerosis is characterized by deposition of immune cells and cholesterol in the subendothelial space of arteries. As EAT is a metabolically endocrine tissue with abundant proinflammatory cytokines, it is considered to be associated with atherosclerosis. It is reported that EAT leads to CVDs by involving in the mechanism of inflammation, insulin resistance, and oxidative stress. C1q TNF-related protein 3 (CTRP3) is an adipokine with anti-inflammatory and cardioprotective properties. It has also been demonstrated to activate nuclear factor kappa B (NF-κB) signaling and PI3K/Akt/eNOS pathway to attenuate obesity-related inflammation responses and insulin resistance and thus regresses atherosclerosis (52, 53). A study involving 34 patients with elective post-coronary artery by-pass graft (post-CABG) showed that EAT with lower CTRP3 mRNA level is closely associated with coronary atherosclerosis and cardiac dysfunction (54). IL-1β and angiopoietin-like-4 (an inhibitor of lipoprotein lipase secreted by adipose tissue) were highly expressed in EAT patients with coronary artery diseases (55). Adipocyte oxidative stress, characterized by the imbalance of ROS and redox signaling, is related to metabolic CVDs. It has been demonstrated that EAT can produce more ROS compared to subcutaneous adipose tissue because it has higher expression of NADPH components gp91phox and p47 phox (56). The hyperglycemia and insulin resistance accelerate adipocyte oxidative stress (57–59). A recent study showed that patients with severe coronary atherosclerosis, glucose and insulin metabolic disorder, and serum adiponectin reduction are significantly linked with higher oxidative stress in EAT adipocytes (60). In addition, EAT thickness was related with endothelial dysfunction (61), and it was concluded that EAT may predict the early reversible stages of atherosclerosis. The data from different studies showed that increased EAT volume is involved in high-risk coronary plaque formation. In addition, patients with high-risk coronary plaque have quantitatively higher EAT volume (35, 62). A prospective cohort study found that EAT and plasma IL-8 level are associated with elevated coronary artery calcium score, which is an independent predictor of coronary atherosclerosis (63).

EAT functions as an endocrine organ. Recent studies have focused on the signaling molecules released by EAT. A novel mechanism for EAT-induced CVDs is the secretion of exosomes that contain non-coding RNAs, especially microRNAs (miRNAs), which are subsequently absorbed by endothelial cells or cardiomyocytes (64–66). A previous study verified that increased has-miRNA-34a is associated with coronary artery diseases (67). A recent micro- and lncRNA microarrays followed by GO-KEGG functional enrichment analysis demonstrated a sex-dependent unique mi/lncRNAs. They are involved in inflammation, adipogenesis, and cardiomyocyte apoptosis. They are also modified in human epicardial fat in both patients with and without coronary artery disease. Examples include has-miR-320 family, hsa-miR-21, hsa-24-3p, hsa-miR-378, and hsa-miR-33 (68).

### 4.2 Arrhythmias

Atrial fibrillation (AF), the most common arrhythmia, is the major cause of ischemic stroke, heart failure, and cardiovascular mortality. Atrial electrophysiological and structural remodeling is the underlying mechanism of AF, which is characterized by myocardial fibrosis, and the underlying mechanism is heterogeneous (69). The mechanism of arrhythmias includes adipocyte infiltration, pro-fibrotic, and pro-inflammatory paracrine effects, oxidative stress, and other pathways (69–71). A study in 215 acute embolic stroke patients showed that increased periatrial EAT thickness on the left side is associated with AF (72). In patients with AF who have undergone pulmonary vein isolation, EAT volume is associated with AF recurrence (73). In another study, most of the persistent AF patients who are not anticoagulated and with increased periatrial EAT thickness were also associated with an increased risk of cardiovascular events (74). Julia and colleagues found that patients with lone AF have larger volume and higher attenuation of EAT compared with patients without cardiac arrhythmias (75). Moreover, non-uniformity of EAT radiomic gray level is the only independent predictor of post-ablation AF recurrence within 12 months follow-up (75).

EAT was found to be significantly higher in the patients with nephrotic syndrome and in patients with ECG showing the atrial depolarization and ventricular repolarization (76). Another study demonstrated that EAT volume exerts reverse relationship with heart rate recovery that indicates the potential adverse effects of EAT on cardiac autonomic function (77). It may result from the pathogenic effect of local inflammatory cytokines secreted from nearby visceral fats. As an endocrine organ, EAT influences adjacent myocardium by secreting a series of bioactive molecules, such as exosomes carrying circular RNAs (circRNAs), and regulates atrial electrical and structural remodeling. Zheng and colleagues identified differently expressed circRNA in EAT *via* RNA sequencing, such as hsa_circRNA_000932 and hsa_circ_0078619, which may work as endogenous RNAs to capture various miRNAs miR-103a-2-5p and miR-199a-5p, and subsequently regulate the expression of cardiovascular disorders-related protein-coding genes (78).

### 4.3 Aortic stenosis

With the global epidemiological increase in elderly population, AS becomes a challenging disease, representing an important cause of morbidity, hospitalization, and death in aged population. Generally, AS is considered as the result of a complex process, driven by inflammation and involving multifactorial pathological mechanisms promoting valvular calcification and valvular bone deposition (79). Importantly, obesity-related chronic systemic inflammation is associated with a significant increase in the amount of EAT, the cardiac visceral fat, which is considered a transducer of the adverse effects of systemic inflammation and metabolic disorders on the heart (80). As EAT can mediate the deleterious effects of systemic inflammation on the myocardium, it may contribute to the pathogenesis of calcific AS. Parisi et al. hypothesized that EAT may participate in the inflammatory burden of aortic stenosis (81). Mahabadi et al. found that EAT thickness, quantified using transthoracic echocardiography, was significantly associated with severe aortic stenosis, independent of traditional risk factors (82). Moreover, Arangalagea et al. showed that EAT volume was independently associated with LV mass in a prospective cohort of patients with aortic stenosis (83). These results support the hypothesis of a potent proinflammatory activation of EAT in patients with AS and suggest the involvement of cardiac visceral fat in inflammatory and atherogenic phenomena occurring in the AV and promoting its degeneration and calcification (79, 81).

### 4.4 Thromboembolism

The relationship between AF and thromboembolism is well established. Recently, preliminary investigations demonstrate the possible relationship between EAT and AF-related thromboembolism. In a recent study in AF patients who developed stroke, EAT volume was significantly increased. Hence, total EAT is considered as an independent predictor of higher risk of stroke occurrence after AF diagnosis (84). In addition, EAT thickness was higher in non-valvular AF patients compared to healthy subjects. EAT thickness was also related with the CHA2DS2−VASc score in patients with non-valvular AF (85). A multicenter study in Korea enrolled 3,464 individuals and showed that larger peri-atrial EAT volume was independently associated with post-ablation embolic stroke regardless of AF recurrence and CHA2DS2-VASc score (86). Patients with post-ablation embolic stroke had a greater prevalence of prior thromboembolism, lower creatinine clearance, larger left atrial diameter, frequent AF recurrence, and abundant total and peri-atrial EAT (86).

However, another study showed that EAT thickness was directly related with CHA2DS2-VASc scores in patients with sinus rhythm (87). A single-center retrospective study enrolled 202 patients and showed that a thickened EAT was associated with low left atrial appendage flow velocity and had increased risk of thromboembolic phenomena in the presence of AF (88). The mechanism of correlation between EAT and embolic stroke might be explained by EAT-mediated atrial cardiomyopathy, which is characterized by LA enlargement, increased wall stiffness, hypercontractility, endothelial dysfunction, and impaired reservoir function that lead to atrial prothrombotic milieu (84, 86, 89, 90).

### 4.5 Heart failure

EAT is associated with risk factors for HF, such as obesity, metabolic syndrome, hypertension, and diabetes. Numerous studies focused on the relationship between EAT and HF. A study enrolled 72 type-2 diabetes subjects with normal cardiac function and verified that subjects with higher EAT thickness showed a lower cardiac workload, worse cardiopulmonary function and subclinical cardiac systolic dysfunction after maximal cardiopulmonary exercise test with similar duration of exercise (91). Another study found that HF patients have higher EAT than the control group. Hence, EAT can be considered as a prognostic predictor of HF with preserved ejection fraction (HFpEF) (92). In a prospective multinational PROMIS-HfpEF cohort, increased EAT has been shown to be associated with cardiac structural alterations, adiposity, inflammation, lower insulin sensitivity, and endothelial dysfunction related to HFpEF pathology (93). In addition, a proteomic analysis of EAT from 2,416 HFpEF patients found that EAT proteins such as CD36, POSTN, and TRAP1 were differentially expressed in HFpEF (94). In another study, increased EAT thickness was found closely related with brachial–ankle pulse-wave velocity in HFpEF patients and indicated that thicker EAT may be independently associated with arterial stiffness (95).

Interestingly, in a multicenter cohort study, EAT thickness was found to be greater in patients with HFpEF than HFrEF/HFmrEF. In addition, the EAT thickness is associated with reduced left atrial and left ventricle function in HFpEF, but with better function in HFrEF/HFmrEF (96). Similar results were also found in post-ablation AF patients (97). However, in patients with non-ischemic cardiomyopathy, EAT volume was found to be greater in the LV reverse remodeling group than in the non-remodeling group, which suggests that EAT volume is an independent predictor of LV reverse remodeling in patients with non-ischemic cardiomyopathy (98). Findings by Hao and colleagues indicate that EAT mediates cardiomyocyte apoptosis after acute myocardial infarction through secretion of complement factor D and activation of poly ADP-ribosepolymerase-1 (99), which may subsequently result in heart failure. A recent study in mice model with preserved ejection fraction found that inflammasome-mediated pyroptosis pathway was activated in the EAT. Moreover, suppression of pyroptosis-related protein gasdermin D in cultured EAT could lower cardiomyocyte inflammation and autophagy (100).

### 4.6 COVID-19-related cardiac syndrome

The coronavirus disease 2019 (COVID-19) pandemic has spread worldwide with more than 6 million deaths recorded globally (101). Besides pneumonia, myocardial injury is a typical COVID-19-related complication and is present in 20%–30% of patients that contributed to 40% of deaths (102). Patients with larger EAT seem to get higher cardiac risk in COVID-19 patients (103, 104), with worse outcomes (105). Moreover, the type of adipose tissue and its distribution seems to play a crucial role in COVID-19 severity (101). It is well-known that EAT has direct anatomical and functional contiguity with the myocardium, and these two tissues share the same microcirculation, which support the pathophysiology of COVID-19-related cardiac injury. Therefore, clinical studies and practice on COVID-19-related CVDs have focused on cardiac adipose tissue. Recent studies have shown that in patients with COVID-19, higher EAT volume and lower EAT density may be independent predictors of an unfavorable disease prognosis, including cardiovascular complications and death (104, 106, 107). It is reported that EAT is like a highly inflammatory region with dense macrophage infiltrates and highly enriched proinflammatory cytokines, which are overexpressed in COVID-19 patients with CVDs that facilitates viral spread and augments immune response (18, 108, 109). COVID-19-related cardiac injury is characterized by decreased angiotensin-converting enzyme 2 (ACE2) and entry ligand receptor, with pathogenetic role (20, 108). Previous studies indicated that ACE2 deficiency mediates myocardial inflammation, and ACE2 reduction is associated with EAT inflammation (110). In addition, ACE2 downregulation leads to the proinflammatory polarization of M1 macrophages in EAT and results in the dysregulation of the inflammatory response, which is highly observed in COVID-19. Moreover, ICU patients with a higher EAT volume had a higher risk of developing pulmonary embolism compared to those with lower EAT volume (111). Therefore, EAT plays a role in COVID-19-related CVDs and has potential to become a clinically measurable and modifiable therapeutic target.

## 5 Therapeutic options in EAT

**Figure 3:**
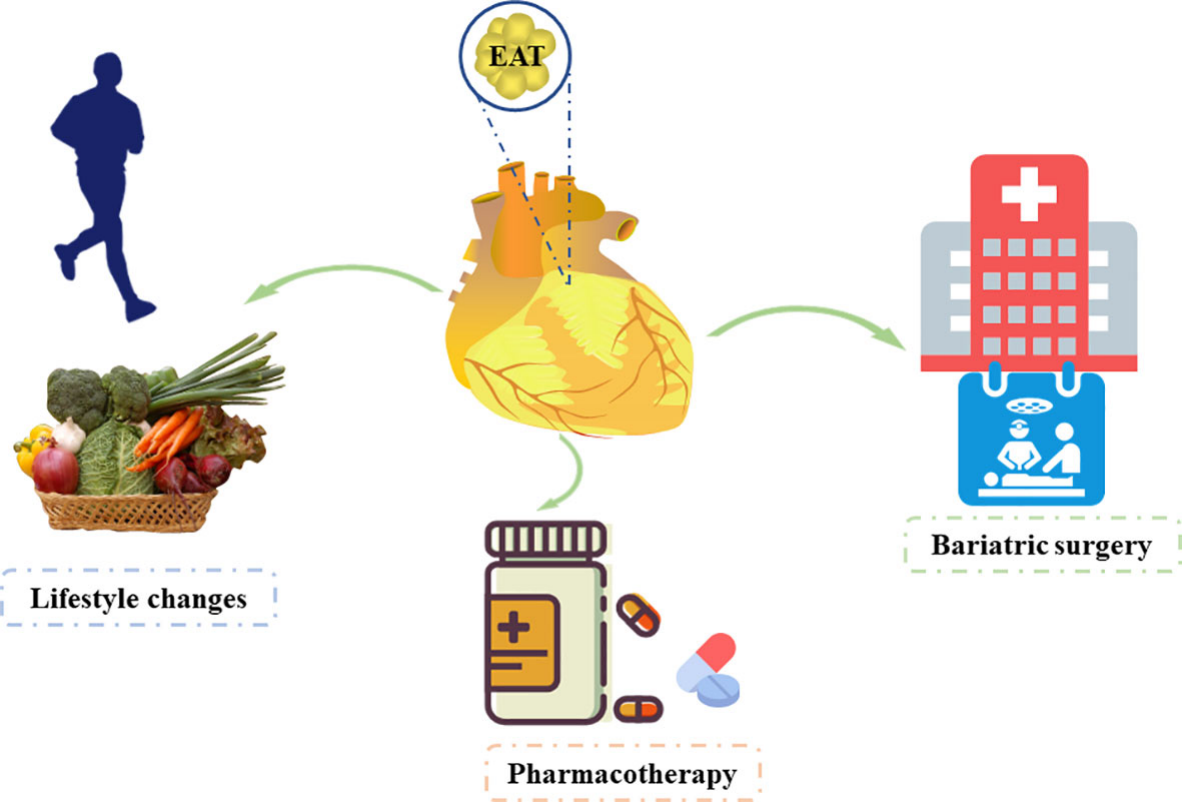
Therapeutic options affecting the epicardial adipose tissue.

We have discussed that EAT is an independent risk factor and has potential to become a therapeutic target for CVDs. Hence, studies have focused on reducing EAT (**Figure 3**).

### 5.1 Lifestyle intervention

EAT is exacerbated by several unhealthy life styles, such as sedentary life, weight gain, and an unbalanced diet (112, 113). Lifestyle intervention based on dietary control and regular physical activity is an essential “first-line” strategy for the clinical management of obesity. Physical exercise and strict diet control reduce visceral fat, including EAT, and improve cardiac function (114, 115). A recent study showed that bad childhood experience is associated with increased EAT in children with depression and reduced physical exercise (116). Studies have indicated that regular physical activity is an effective non-invasive strategy for reducing EAT that may provide beneficial effects on the cardiovascular system (113, 117, 118). Several studies showed that aerobic exercise decreases EAT thickness significantly in obese men (118, 119). Another study from India showed that the 12-week regular Taekwondo training reduces the EAT thickness significantly in elderly women with hypertension (120). Another study from Turkey enrolled 74 obese women and found that long-term, sustained weight loss reduces epicardial fat thickness significantly as assessed by echocardiography, which can be used as an indicator of metabolic profile for weight reduction in obese women (121). Moreover, the decrease in epicardial fat thickness was significantly higher in patients who reversed their metabolic syndrome diagnosis with weight loss than in those whose metabolic syndrome status was unchanged. Iacobellis et al. reported significant reduction in epicardial fat in severely obese patients after 6 months of low-calorie diet (122). However, a systematic review and meta-analysis conducted by Rabkin and Campbell (123) showed that diet and bariatric surgery markedly reduced EAT, but this was not achieved with exercise. Moreover, a reduction in body mass index was significantly associated with reduced EAT by diet-based interventions.

### 5.2 Medical treatment

The impact of medical treatment on EAT is worth investigating. The use of statin is associated with decreased adipokine release from visceral EAT.

Parisi et al. (124) reported that statin therapy was significantly associated with lower EAT thickness and with lower levels of EAT-secreted inflammatory mediators. Of note, there was a significant correlation between EAT thickness and its proinflammatory status. Among lipid-lowering agents, atorvastatin has more significant effect than simvastatin and ezetimibe (125). Other studies also demonstrated that statins reduce EAT volume (124, 126). Additionally, antidiabetic drugs such as GLP-1A (127, 128), metformin (129, 130), and SGLT2 inhibitors (131, 132) were also proved to reduce EAT. For individuals with severe obesity, bariatric surgery is the most reliable treatment. It is well-known that different depots of adipose tissue and visceral fat change after bariatric surgery. Weight loss following bariatric surgery is associated with EAT reduction (133). Hunt et al. reported that severely obese subjects have lower EAT during a 14-year follow-up after bariatric surgery (134).

## 6 Conclusion

The cardiovascular system is widely affected by EAT. The expansion and remodeling of EAT contributes to vascular dysfunction and CVDs significantly. The evolving field of non-invasive imaging technique-based EAT composition analysis showed great potential for the stratification of CVD risk. Therefore, it is critical to identify strategies that are capable of reducing cardiovascular risk by modulating EAT mass, distribution, and function. At present, there is growing interest regarding EAT. In the future, the assessment of EAT may become part of the clinical practice to help clinicians identify patients at great risk of developing CVDs and to provide information on their clinical and therapeutic prognosis.

## Author contributions

CL reviewed the literature and drafted this review. XL reviewed the literature and corrected the figures. BA, LC, and WL reviewed the literature, gave critical comments, and revised the manuscript. YW gave critical comments and revised the manuscript. YW and HZ reviewed the literature, gave critical comments, revised the manuscript, and took charge of project supervision and administration. All authors contributed to the article and approved the submitted version.
